# Core Body Temperature Negatively Correlates with Whole-Brain Gray Matter Volume: A Pilot Study in the Context of Global Warming

**DOI:** 10.3390/brainsci15121324

**Published:** 2025-12-12

**Authors:** Keisuke Kokubun, Kiyotaka Nemoto, Yoshimitsu Yamamoto, Ayumu Mitera, Yoshinori Yamakawa

**Affiliations:** 1 Graduate School of Management, Kyoto University, Kyoto 606-8501, Kyoto, Japan; 2 Department of Medical Informatics and Management and Psychiatry, Institute of Medicine, University of Tsukuba, Tsukuba 305-8575, Ibaraki, Japan; 3 Mitsufuji Corporation, Kyoto 619-0237, Kyoto, Japan; 4 Institute of Innovative Research, Institute of Science Tokyo, Meguro 152-8550, Tokyo, Japan; 5 ImPACT Program of Council for Science, Technology and Innovation, Cabinet Office, Government of Japan, Chiyoda 100-8914, Tokyo, Japan; 6 Office for Academic and Industrial Innovation, Kobe University, Kobe 657-8501, Hyogo, Japan; 7 Brain Impact, Kyoto 606-8501, Kyoto, Japan

**Keywords:** global warming, core body temperature, gray matter volume (GMV), brain structure, magnetic resonance imaging (MRI), inflammation, oxidative stress, environmental neuroscience, temperature regulation, neural health

## Abstract

Global warming has been associated with various adverse effects on human physiology, yet its potential impact on brain structure remains largely unexplored. The present pilot study investigated the relationship between core body temperature and whole-brain gray matter volume (GMV) in healthy adults. Twenty-seven participants (19 males, 8 females; mean age = 38.6 ± 10.3 years) underwent MRI scanning and core temperature assessment. Correlation and partial correlation analyses were performed to examine the association between core body temperature and GMV, controlling for demographic and physiological covariates summarized by the first principal component. Core body temperature showed a significant negative correlation with whole-brain GMV (r = −0.496, *p* = 0.009; 95% CI = −0.737 to −0.143) and a trend-level significant partial correlation after covariate adjustment (r = −0.373, *p* = 0.060; 95% CI = −0.660 to 0.008). These trends remained after correction for multiple comparisons using the Benjamini–Hochberg false discovery rate. Exploratory analyses across 116 AAL regions identified the left Fusiform gyrus as showing a significant negative correlation with core body temperature (r = −0.643, *p* < 0.001). Given the modest sample size, these findings should be interpreted cautiously as preliminary, hypothesis-generating evidence. They suggest that even subtle variations in body temperature within the normal physiological range may relate to differences in global brain structure. Possible mechanisms include heat-induced inflammation, oxidative stress, and increased metabolic load on neural tissue. Understanding how individual differences in body temperature relate to brain morphology may provide insights into the neural health consequences of rising environmental temperatures.

## 1. Introduction

Global temperatures have risen markedly as human economic activity has intensified. Between 1850 and 2020, near-surface air temperature increased by more than 1.2 °C, with the most rapid warming occurring after 1990 [1]. By the end of this century, global temperatures are projected to rise by up to 5.5 °C relative to pre-industrial levels [2,3]. Paleoclimatic evidence further suggests that Homo species exhibited smaller average brain sizes during historically warmer periods [4], possibly reflecting an adaptive reduction in metabolic heat production, given that the brain consumes substantial energy and generates disproportionate heat relative to its mass [5,6,7].

A fundamental step toward understanding how heat exposure affects gray matter is clarifying the mechanisms that regulate body and brain temperature. The hypothalamus serves as the central thermoregulatory hub, integrating internal and external thermal signals and coordinating autonomic, endocrine, and behavioral responses [8]. Within the preoptic area, warm-sensitive neurons regulate heat-dissipating effectors—such as peripheral vasodilation and sweating—while projections to autonomic centers in the brainstem (e.g., medullary raphe nuclei) modulate sympathetic outflow to brown adipose tissue and cutaneous vasculature [9]. Concurrently, the hypothalamus interacts with cortical and limbic regions through neuroendocrine pathways, including activation of the hypothalamic–pituitary–adrenal (HPA) axis and cortisol release, to support stress-related thermogenesis.

Heat exposure activates these neural and endocrine circuits and simultaneously recruits vascular control centers, increasing skin blood flow and heart rate to facilitate heat loss. Sustained activation of these systems alters cerebral blood flow, metabolic demand, and hormonal profiles. In particular, chronic elevations in glucocorticoids have been linked to dendritic remodeling and volume reductions in temperature-sensitive cortical regions [10]. These upstream regulatory pathways affect cortical tissue indirectly through neuroinflammatory and oxidative processes: heat-induced immune activation promotes cytokine release, vasodilation, and tissue swelling [11,12], while reactive oxygen species damage lipids, proteins, and DNA [13].

The cortex is unlikely to be uniformly vulnerable to such physiological stressors. Layers II/III and V are particularly rich in pyramidal neurons characterized by extensive synaptic arborization and high metabolic demands [14,15]. These layers may be especially sensitive to thermal and inflammatory stressors that impair mitochondrial function, synaptic transmission, or local vascular regulation. Disruption of the blood–brain barrier—an established consequence of elevated brain temperature—may further compromise neurons and glia across these layers.

Consistent with these mechanisms, neuroimaging evidence from heatstroke cases (core body temperature > 40 °C) has documented widespread structural damage across the cerebellum, hippocampus, thalamus, basal ganglia, brainstem, and frontal, temporal, and parietal cortices [16,17,18]. Cohort studies similarly indicate that environmental heat increases the risk for cognitive impairment and dementia-related hospitalizations [19,20,21].

Beyond extreme heat exposure, emerging evidence from large cohorts suggests that long-term exposure to moderate heat also influences brain morphology. In the UK Biobank, the proportion of days exceeding 27 °C over 20 years was associated with reduced gray and white matter volumes and accelerated age-related atrophy [22]. Natural sunlight exposure likewise correlated with reductions in total brain, white matter, and gray matter volumes, particularly among younger adults and during summer months [23].

Mechanistically, sunlight and ambient heat elevate brain temperature via effects on blood temperature and cerebral blood flow [24,25,26]. Even <1 °C increases can alter conduction velocity, resting membrane potentials, and synaptic transmission [27]. Since brain temperature typically exceeds core temperature by 0.5–1 °C [28], localized neuroinflammatory responses may generate even greater regional heat elevations. This is consistent with findings that brain hyperthermia accompanies neuroinflammation, leukocyte extravasation, edema, and blood–brain barrier disruption [29]. Reductions in gray matter volume have also been reported in conditions such as COVID-19, where brain temperature is known to increase [30].

Elevated body temperature is additionally associated with depressive symptoms [31,32], exacerbation of psychiatric symptoms [33], elevated suicide risk [34], and impaired cognitive performance even under moderately warm conditions (>25.7 °C) [35]. These findings collectively suggest that thermal load interacts with neural structure and function through diverse physiological pathways.

Despite increasing recognition of the neural, vascular, endocrine, and inflammatory mechanisms linking heat exposure to the brain, no study has directly examined how individual differences in core body temperature relate to gray matter structure in healthy adults under non-extreme environmental conditions. Investigating this relationship may reveal early, subclinical signatures of heat-related stress on cortical morphology—including potential layer-specific vulnerabilities—before overt pathology emerges.

To address this gap, the present pilot study investigates associations between core body temperature and gray matter volume in a sample of 27 healthy adults.

## 2. Materials and Methods

### 2.1. Participants

To achieve a statistical power of 80% with a significance level of 10% for correlation analysis, the required sample size was estimated using G*Power 3.1.9.7. Based on Cohen’s [36] criterion for a “strong correlation” (effect size r = 0.5), the minimum sample size was calculated to be 21 participants. Following an open call, a total of 29 individuals (19 men and 10 women; mean age = 39.0 ± 10.1 years) were recruited for this study. Data from 27 individuals (17 men and 10 women; mean age = 38.6 ± 10.3 years), excluding two incomplete responses, were used for the analysis.

All participants were assembled at Tokyo University of Science in June 2025, where they completed an online questionnaire and participated in MRI data acquisition. According to self-reports, none of the participants had any history of neurological, psychiatric, or other medical conditions that could affect the central nervous system.

All procedures were conducted in accordance with relevant ethical guidelines and regulations. Written informed consent was obtained from all participants prior to the study, and anonymity was maintained throughout the data collection process. The study protocol was approved by the Ethics Committee of the Brain Informatics Cloud, Tokyo Institute of Science (Human Research Ethics Committee Approval No. 2023137).

### 2.2. MRI Data Acquisition

MRI data were acquired using a 3-Tesla MRI scanner (MAGNETOM Prisma, Siemens, Munich, Germany) equipped with a 32-channel head coil. A three-dimensional (3D) T1-weighted magnetization-prepared rapid acquisition gradient echo (MPRAGE) pulse sequence was employed, along with spin-echo echo-planar imaging (SE-EPI) using generalized autocalibrating partially parallel acquisition (GRAPPA). The imaging parameters were as follows: repetition time (TR) = 1900 ms, echo time (TE) = 2.52 ms, inversion time (TI) = 900 ms, flip angle = 9°, matrix size = 256 × 256, field of view (FOV) = 256 mm, and slice thickness = 1 mm.

### 2.3. MRI Data Analysis

T1-weighted images were processed using Statistical Parametric Mapping software (SPM12 v7771; Wellcome Trust Centre for Neuroimaging, London, UK) implemented in MATLAB R2020b (MathWorks Inc., Sherborn, MA, USA), employing the standard SPM12 tissue probability templates.

The segmented gray matter (GM) images were spatially normalized using diffeomorphic anatomical registration through the exponential Lie algebra (DARTEL) algorithm [37]. The preprocessing pipeline included a modulation step and spatial smoothing with an 8 mm full width at half maximum (FWHM) Gaussian kernel.

The smoothed GM images were then proportionally adjusted by dividing each by the intracranial volume (ICV) to obtain proportional GM images. These were used to compute mean and standard deviation (SD) maps. Using this information and the Automated Anatomical Labeling (AAL) atlas [38], regional GM quotients were averaged to calculate the Gray Matter Brain Healthcare Quotient (GM-BHQ), standardized to have a mean of 100 and an SD of 15. See Nemoto et al. [39] for more details.

We clarify the distinction between GMV and GM-BHQ below. Traditional GMV represents the total or proportional amount of gray matter and is typically treated as a single global volumetric measure. In contrast, GM-BHQ differs in both its computational procedure and interpretive meaning. GM-BHQ is derived by first computing standardized quotients for each of the 116 regions defined by the AAL atlas. Specifically, regional gray matter values are converted to z-scores based on a normative database and then transformed to an IQ-like scale (mean = 100, SD = 15). These regional quotients are subsequently averaged to generate a single global GM-BHQ index.

A key advantage of this regional standardization approach is that it incorporates regional heterogeneity in vulnerability to atrophy. By normalizing each region before averaging, GM-BHQ gives equal weight to all cortical and subcortical areas regardless of their absolute size, thereby providing a more sensitive indicator of overall “brain health” than raw global GMV. In this study, GMV refers to the total absolute gray matter volume calculated by summing all voxel values in the modulated gray matter images without normalization by intracranial volume (ICV).

### 2.4. Core Body Temperature

The pulse-to-pulse interval (PPI) was obtained from the wrist using an optical sensor embedded in the smartwatch MITSUFUJI 03 (https://www.mitsufuji.co.jp/mitsufuji03/, accessed on 1 December 2025), and the deep body temperature was estimated according to the algorithm developed by Kurosaka et al. [40]. The algorithm for estimating changes in core body temperature has been patented in Japan by the University of Occupational and Environmental Health, Maeda Corporation, and Mitsufuji (Japanese Patent No. 7175473: Core Body Temperature Estimation Device, Method, and Program).

### 2.5. Steps, Stress Level, and Ambient Temperature

Data on steps, stress level, and ambient temperature were collected using the same smart watch MITSUFUJI 03. Steps were calculated using the device’s GPS function. Stress level was estimated from the same photoplethysmography (PPI) signal used to derive core body temperature, by computing the LF/HF ratio (an index of sympathetic–parasympathetic balance) and applying a proprietary algorithm. Ambient temperature was measured as the environmental temperature around the watch band.

### 2.6. Data Analysis

Correlation and partial correlation analyses were conducted to examine the relationship between core body temperature and whole-brain GMV. Given that this was a small-sample pilot study, statistical “trend-level” significance was defined as *p* < 0.1. This criterion was selected because the primary purpose of a pilot study is to obtain preliminary information that can guide future large-scale research; therefore, avoiding Type II errors was prioritized over minimizing Type I errors. This decision is consistent with previous work [41], and several recent pilot studies have similarly adopted a threshold of *p* < 0.1 [42,43,44]. Nonetheless, we explicitly distinguished this trend-level criterion from the conventional significance threshold of *p* < 0.05.

To control for multiple comparisons, both correlation and partial correlation analyses were corrected using the Benjamini–Hochberg false discovery rate method. Correlation coefficients were interpreted using Cohen’s [36] effect size conventions: r = 0.5 was considered a “strong” correlation, whereas r = 0.3 represented a “medium” correlation.

Partial correlations were adjusted using principal component scores that captured the shared variance across a broad set of potentially confounding variables. These included study date, study time, age, BMI, systolic and diastolic blood pressure, pulse rate, average daily step count, self-reported stress, and ambient environmental temperature. This dimensionality-reduction approach was adopted to maximize degrees of freedom in the small sample while adequately accounting for multiple physiological, behavioral, and environmental factors known to influence body and brain temperature [45].

Although the primary analyses used a trend-level significance threshold (*p* < 0.1) due to the pilot nature of the study, we additionally conducted exploratory correlations across 116 AAL regions using a more conservative threshold of *p* < 0.001. All statistical analyses were performed using IBM SPSS Statistics, Version 28 (IBM Corp., Armonk, NY, USA).

## 3. Results

**Figure 1:**
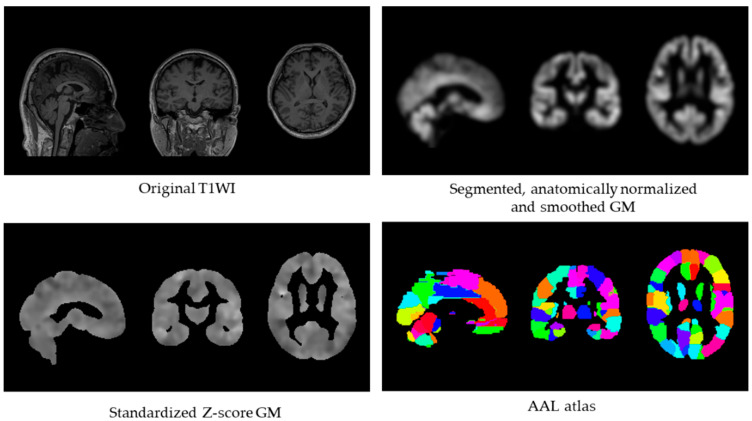
GM-BHQ calculation pipeline. Processing steps from original T1-weighted image (**top left**) to segmented, normalized, and smoothed gray matter (**top right**), standardized Z-score image (**bottom left**), and regional extraction using the AAL atlas with 116 regions (**bottom right**). The GM-BHQ is computed as the average of regional quotient values.

Figure 1 illustrates the computational pipeline used to derive the GM-BHQ metric.

Table 1 presents the descriptive statistics. Principal component analysis revealed that the scree plot exhibited eigenvalues of 3.000, 1.793, 1.537, 1.416, and 1.032, with a clear inflection between the first factor (explaining 27.27% of the variance) and the second. This pattern indicates that a one-factor solution provides the most appropriate representation of the shared variance among the control variables. Therefore, to preserve degrees of freedom in the regression model, we used the first principal component score of the demographic and physiological variables—as shown in Table 2—as the covariate, rather than including each variable individually.

Core body temperature showed a significant negative correlation with whole-brain GMV (r = −0.496, *p* = 0.009; 95% CI = −0.737 to −0.143) and a marginally significant partial correlation after controlling for demographic, physiological, and environmental covariates (r = −0.373, *p* = 0.060; 95% CI = −0.660 to 0.008). The former represents the unadjusted association, whereas the latter reflects the correlation after accounting for potential confounders. In terms of effect size, both coefficients fall between Cohen’s [36] conventions for a medium effect (r = 0.3) and a large effect (r = 0.5). Both trend-level associations (*p* < 0.1) remained after correction for multiple comparisons using the Benjamini–Hochberg false discovery rate procedure.

In an exploratory analysis across 116 AAL regions using a conservative threshold of *p* < 0.001, only the left fusiform gyrus exhibited a significant negative correlation with core body temperature (r = −0.643, *p* < 0.001; 95% CI = −0.822 to −0.348). In the corresponding partial correlation analysis controlling for the first principal component, no regions met the *p* < 0.001 criterion; however, the left fusiform gyrus again showed the strongest association (r = −0.571, *p* = 0.002; 95% CI = −0.781 to −0.243). Both coefficients exceeded the threshold for a large effect size according to Cohen [36].

Given the small sample size (n = 27), the statistical power of this study is necessarily limited, particularly for detecting the medium effect size observed in the adjusted whole-brain GMV analysis. Power considerations suggest that the current sample is adequate for identifying medium-to-large effects but may fail to detect smaller associations. Accordingly, the present findings should be interpreted as preliminary and in need of replication in larger and more diverse cohorts.

**Figure 2:**
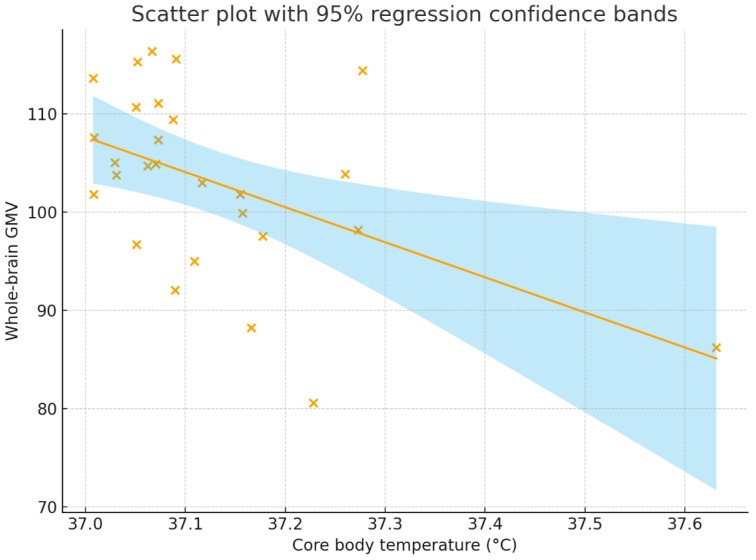
Correlation between core body temperature (°C) and whole-brain GMV. The correlation coefficient was r = −0.496, *p* = 0.009, with a 95% confidence interval of [−0.737, −0.143]. The solid line represents the regression line, the shaded region represents the 95% regression confidence bands, and each “×” indicates an individual participant’s data point. GMV = gray matter volume.

**Figure 3:**
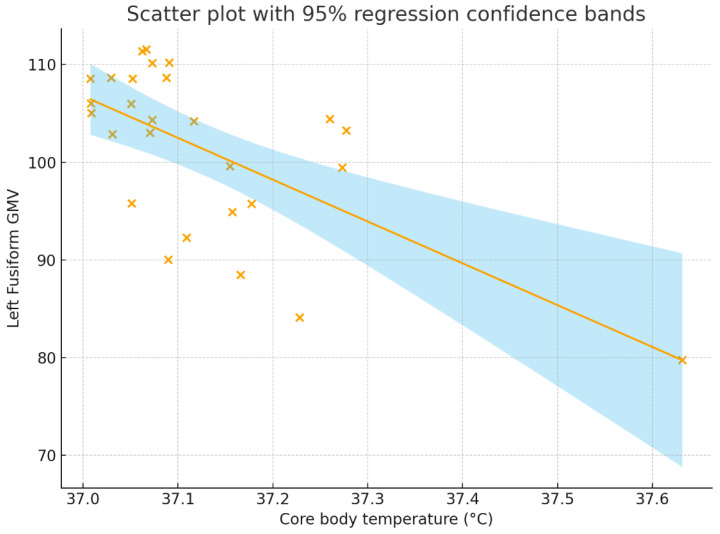
Correlation between core body temperature (°C) and whole-brain GMV. The correlation coefficient was r = −0.643, *p* < 0.001, with a 95% confidence interval of [−0.822, −0.348]. The solid line represents the regression line, the shaded region represents the 95% regression confidence bands, and each “×” indicates an individual participant’s data point. GMV = gray matter volume.

Figure 2 and Figure 3 present a scatter plot illustrating the association between core body temperature and whole-brain and the left fusiform GMV.

To evaluate the robustness of these associations, we reanalyzed the data after removing one participant whose core body temperature exceeded 37.6 °C and therefore represented a potential physiological outlier. After excluding this data point, the negative correlation between core body temperature and whole-brain GMV remained statistically significant (r = −0.463, *p* = 0.017; 95% CI = −0.737 to −0.143), and the corresponding partial correlation controlling for demographic, physiological, and environmental covariates continued to show a trend-level effect (r = −0.293, *p* = 0.155; 95% CI = −0.617 to 0.115).

For the left fusiform gyrus, the robustness analysis similarly reproduced the original findings. The simple Pearson correlation remained significant (r = −0.463, *p* = 0.017; 95% CI = −0.737 to −0.143), indicating that higher core body temperature was associated with reduced local GMV. When controlling for demographic, physiological, and environmental covariates, the partial correlation continued to show a moderate negative association (r = −0.415, *p* = 0.039; 95% CI = −0.696 to −0.024). Notably, both coefficients remained within the medium-to-large effect range according to Cohen’s [36] benchmarks. The consistency of the direction and approximate magnitude of these coefficients across both whole-brain and regional analyses suggests that the observed brain–temperature associations were not attributable to a single extreme value but instead reflect robust interindividual variation.

## 4. Discussion

The present study demonstrated a significant negative correlation between core body temperature and whole-brain GMV in healthy adults, which remained at a trend level after adjusting for demographic and physiological covariates. To our knowledge, this is the first evidence suggesting that even subtle variations in core body temperature within the normal physiological range are associated with differences in global brain structure.

Previous research has focused primarily on extreme heat exposure, such as in patients with heatstroke or populations living in high-temperature environments, showing that elevated body temperature can induce neuronal damage, inflammation, and brain atrophy [12,13]. Environmental and epidemiological studies similarly report that higher ambient temperatures and prolonged sunlight exposure are associated with reduced GMV, increased dementia-related hospitalizations, and cognitive decline [19,20,23]. Importantly, recent evolutionary work suggests that larger brains impose substantial energetic and thermoregulatory costs, which may become increasingly burdensome in warmer climates [46]. This perspective provides a complementary lens for interpreting our findings: if bigger or metabolically active brains require greater heat dissipation, even modest elevations in core body temperature might disproportionately strain neural tissue. Our results extend these observations by suggesting that small, everyday variations in body temperature among healthy individuals may likewise influence brain volume.

One plausible mechanism involves thermally induced inflammatory and oxidative processes. Heat exposure activates inflammatory mediators and reactive oxygen species, which can disrupt the blood–brain barrier, impair mitochondrial function, and lead to neuronal injury [11,27]. Localized increases in brain temperature have also been linked to vascular leakage, edema, and tissue damage [14,29]. Even modest increases in deep body temperature may therefore contribute to microstructural or volumetric changes in gray matter over time.

A second mechanism pertains to thermoregulatory efficiency and cerebral metabolism. The brain is metabolically demanding and produces substantial heat relative to its mass [5,6]. Individuals with higher baseline core temperatures may experience greater metabolic strain, necessitating more intensive thermoregulatory processes to maintain homeostasis. These sustained demands could, over time, influence neuronal integrity or glial function, thereby contributing to the volumetric patterns observed in the present study.

In exploratory analyses across 116 AAL regions, only the left fusiform gyrus showed a significant negative correlation with core body temperature (*p* < 0.001). Although this result must be interpreted cautiously due to the limited sample size and lack of full statistical control, it nonetheless provides a physiologically plausible hypothesis for future investigation. The hypothalamus is the central thermoregulatory hub, integrating autonomic and neuroendocrine signals, including those of the HPA axis [47,48,49,50]. Through its regulation of sympathetic output, the hypothalamus can alter local vascular reactivity, which in turn may affect metabolic supply and cellular stress in specific cortical regions such as the fusiform gyrus.

HPA axis activation and associated fluctuations in glucocorticoid levels may further contribute to region-specific structural modulation. Chronic or repeated glucocorticoid exposure produces transcriptional effects via mineralocorticoid (MR) and glucocorticoid receptors (GR), whose distribution varies by cell type and cortical region [51,52]. From a laminar perspective, the cerebral cortex consists of six layers (I–VI), with superficial layers (I–III) particularly rich in input and local circuit activity. Thermal stress and prolonged HPA activation may preferentially affect neurons and synapses in these superficial layers through vascular and metabolic mechanisms, potentially leading to subtle cortical structural changes. Within this conceptual framework, the left fusiform association observed in this study may reflect the cumulative influence of several interacting pathways:

1. Variations in core body temperature → altered hypothalamic activity.
2. Changes in autonomic (sympathetic) output → fluctuations in local vascular reactivity.
3. HPA axis modulation → alterations in cortisol secretion.
4. Layer-specific cortical responses (I–III) mediated by vascular, metabolic, and GR/MR-dependent transcriptional processes → subtle structural effects on fusiform GMV.

**Figure 4:**
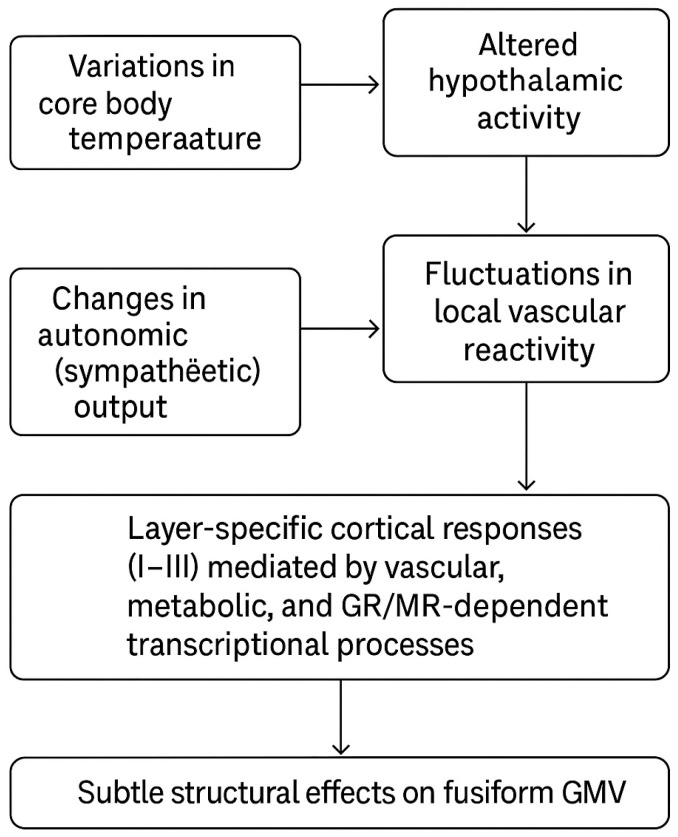
Multilevel physiological pathways linking core body temperature to fusiform gray matter volume. This figure is original and created by the authors.

The fusiform gyrus is a critical region involved in visual and semantic processing. In semantic dementia, for example, left fusiform atrophy has been linked to semantic deficits, and fusiform degeneration contributes to impairments observed in Alzheimer’s disease and prosopagnosia [47]. It is conceivable that heat-related structural vulnerability may emerge initially in regions such as the left fusiform gyrus and, with progressive cognitive decline, extend toward more widespread cortical atrophy—an intriguing hypothesis for future longitudinal research (Figure 4).

Importantly, the present study was conducted with a modest sample (n = 27), which limits both statistical power and the generalizability of the findings. Although the G*Power calculations indicated adequate power to detect large effects (r > 0.5), the observed partial correlation (r = −0.373) suggests that the study may have been underpowered to reliably detect medium-sized associations. Accordingly, the results should be interpreted as preliminary and hypothesis-generating rather than definitive.

Furthermore, the controlled laboratory context and small, convenience-based sample mean that these findings should not be extrapolated to population-level environmental or climatic effects. Most prior work showing cognitive or neural consequences of heat exposure has examined short-term, acute physiological responses over hours or days, whereas global warming unfolds over decades or longer. The present results therefore do not imply that rising ambient temperatures will directly or proportionally translate into long-term structural changes in the human brain. Instead, they offer a narrow, biologically grounded observation—that individual differences in deep body temperature, measured at a single time point under non-extreme conditions, are detectable in relation to gray matter volume. This preliminary signal motivates, but does not substitute for, future research designed explicitly to bridge short-term physiological mechanisms with long-term environmental exposures.

Future research should include larger, well-powered samples and longitudinal designs to determine whether sustained variations in body temperature causally influence gray matter structure. Incorporating high-resolution MRI for layer-specific cortical thickness, perfusion fMRI to quantify vascular dynamics, autonomic indices (e.g., heart rate variability), and HPA axis biomarkers (e.g., cortisol, ACTH) will be essential to elucidating the interplay between thermoregulation, stress physiology, and cortical microstructure.

### Limitations and Future Directions

This study has several limitations that should be acknowledged. First, the sample size was relatively small. Although this is appropriate for a pilot investigation, it limits the statistical power and generalizability of the findings. Future studies with larger and more diverse samples are needed to validate and refine the observed relationship between body temperature and GMV.

Second, the cross-sectional design precludes causal inference. It remains unclear whether higher body temperature directly contributes to reduced GMV, or whether both are influenced by underlying physiological or environmental factors such as metabolic rate, sleep quality, or circadian rhythms. Longitudinal or experimental studies that manipulate thermal exposure would help clarify these causal pathways.

Third, core body temperature was measured at a single time point. Because body temperature fluctuates according to circadian cycles, physical activity, hydration, and environmental conditions, repeated or continuous measurements would yield more reliable indices of individual thermal profiles.

Fourth, the issue concerns the measurement method. When the smartwatch band is worn loosely, the pulse wave cannot always be captured accurately. Similarly, vigorous movements may also lead to inaccurate pulse-wave acquisition. Therefore, the adopted algorithm removes noise from the obtained pulse-wave signal and corrects the data to estimate changes in core body temperature. Nevertheless, some discrepancies from measurements obtained using direct thermometric or ingestible probe methods may remain, which could have exerted a slight influence on the reported correlation coefficients.

Fifth, although multiple covariates were included, they may not have fully captured all relevant sources of variation. Incorporating more comprehensive multimodal physiological data (e.g., skin temperature, inflammatory markers, medical history, menstrual cycle), socioeconomic variables (e.g., education, occupation), lifestyle information (e.g., hydration status, sleep quality), and detailed environmental exposure indices (e.g., heat index) would further strengthen interpretability.

Lastly, MRI-based volumetric analyses capture macroscopic structural differences but do not directly reflect underlying cellular or molecular mechanisms. Combining structural MRI with complementary modalities—such as diffusion imaging, magnetic resonance spectroscopy, or PET—may help elucidate the specific neurobiological processes through which thermal factors influence brain integrity.

Despite these limitations, the present findings highlight a previously unexplored link between deep body temperature and global brain structure in healthy adults. This relationship may represent a key physiological pathway through which environmental heat stress contributes to neurobiological vulnerability in a warming world.

## 5. Conclusions

In summary, this pilot study demonstrated that higher core body temperature is associated with lower whole-brain gray matter volume in healthy adults, with a trend-level association persisting after adjustment for demographic and physiological covariates. Exploratory analyses further identified the left fusiform gyrus as a region showing a significant negative correlation with core body temperature. Although these findings should be interpreted cautiously given the modest sample size, they suggest that even minor variations in body temperature within the normal physiological range may relate to differences in global and regional brain structure.

These results introduce a new perspective on how physiological thermal load may contribute to neural vulnerability, potentially through mechanisms involving inflammation, oxidative stress, or increased metabolic demand on neural tissue. As global temperatures continue to rise, understanding how individual differences in body temperature relate to brain morphology becomes increasingly important. By showing that core body temperature—a simple and easily measurable physiological index—is linked to brain volume, this study underscores the relevance of environmental temperature as a potential determinant of neural health. Such insights may help inform future research priorities and contribute to public awareness of the broader health implications of a warming climate.

## Figures and Tables

**Table 1:** Descriptive Statistics of Demographic, Physiological, and Environmental Variables.

Variable	Minimum	Maximum	Mean	SD
Study date	3 June 2025	6 June 2025	4 June 2025	1 day 3:33:52
Study time (hh:mm)	9:25	16:48	12:56	2:23
Age (years)	22.0	58.0	38.6	10.3
BMI (kg/m^2^)	16.4	31.2	23.9	3.8
Systolic BP (mmHg)	82.0	146.0	113.6	16.3
Diastolic BP (mmHg)	43.0	99.0	71.9	14.7
Pulse (beats/min)	62.0	114.0	75.6	12.7
Daily steps (mean)	2265.6	10,100.6	5695.3	2076.5
Stress score	19.6	42.5	30.6	6.1
Ambient temperature (°C)	26.5	29.9	27.7	0.8
Core body temperature (°C)	37.0	37.6	37.1	0.1
Whole-brain GMV	80.6	116.4	103.1	9.3

SD: standard deviation. BMI: body mass index. GMV: gray matter volume.

**Table 2:** Factor Loadings From the Principal Component Analysis.

Variable	Factor 1	Factor 2	Factor 3	Factor 4
Study date	0.032	0.615	0.504	−0.214
Study time	−0.319	0.346	−0.650	0.374
Age (years)	0.625	0.248	−0.233	0.241
BMI (kg/m^2^)	0.847	0.088	−0.320	0.085
Systolic BP (mmHg)	0.860	−0.279	0.087	0.082
Diastolic BP (mmHg)	0.830	−0.003	0.132	−0.107
Pulse (beats/min)	0.352	0.582	−0.503	−0.229
Daily steps (mean)	0.033	0.113	0.323	0.718
Stress score	−0.169	0.703	0.261	0.466
Ambient temperature (°C)	0.348	0.379	0.464	−0.285
Sex (male = 1, female = 0)	0.296	−0.398	0.197	0.529

BMI: body mass index.

## Data Availability

The data presented in this study are available on request from the corresponding author due to the need to protect the privacy of participants.
